# Early-Life Exposure to Outdoor Air Pollution and Respiratory Health, Ear Infections, and Eczema in Infants from the INMA Study

**DOI:** 10.1289/ehp.1205281

**Published:** 2012-12-05

**Authors:** Inmaculada Aguilera, Marie Pedersen, Raquel Garcia-Esteban, Ferran Ballester, Mikel Basterrechea, Ana Esplugues, Ana Fernández-Somoano, Aitana Lertxundi, Adonina Tardón, Jordi Sunyer

**Affiliations:** 1 Centre for Research in Environmental Epidemiology (CREAL), Barcelona, Spain; 2 Consortium for Research in Epidemiology and Public Health (CIBERESP), Spain; 3 National Institute of Health and Medical Research (INSERM), U823, Team of Environmental Epidemiology applied to Reproduction and Respiratory Health, Grenoble, France; 4 Centre of Public Health Research (CSISP), Valencia, Spain; 5 School of Nursing, University of Valencia, Valencia, Spain; 6 Public Health Division of Gipuzkoa, Basque Country Government, Gipuzkoa, Spain; 7 BioDonostia Health Research Institute, Donostia, Spain; 8 University of Oviedo, Oviedo, Spain; 9 Department of Public Health and Preventive Medicine, University of Basque Country, Bilbao, Spain; 10 Hospital del Mar Medical Research Institute (IMIM), Barcelona, Spain; 11 Pompeu Fabra University, Department of Experimental and Health Sciences, Barcelona, Spain

**Keywords:** air pollution, children’s health, ear infections, eczema, *in utero* exposure, respiratory infections

## Abstract

Background: Prenatal and early-life periods may be critical windows for harmful effects of air pollution on infant health.

Objectives: We studied the association of air pollution exposure during pregnancy and the first year of life with respiratory illnesses, ear infections, and eczema during the first 12–18 months of age in a Spanish birth cohort of 2,199 infants.

Methods: We obtained parentally reported information on doctor-diagnosed lower respiratory tract infections (LRTI) and parental reports of wheezing, eczema, and ear infections. We estimated individual exposures to nitrogen dioxide (NO_2_) and benzene with temporally adjusted land use regression models. We used log-binomial regression models and a combined random-effects meta-analysis to estimate the effects of air pollution exposure on health outcomes across the four study locations.

Results: A 10-µg/m^3^ increase in average NO_2_ during pregnancy was associated with LRTI [relative risk (RR) = 1.05; 95% CI: 0.98, 1.12] and ear infections (RR = 1.18; 95% CI: 0.98, 1.41). The RRs for an interquartile range (IQR) increase in NO_2_ were 1.08 (95% CI: 0.97, 1.21) for LRTI and 1.31 (95% CI: 0.97, 1.76) for ear infections. Compared with NO_2_, the association for an IQR increase in average benzene exposure was similar for LRTI (RR = 1.06; 95% CI: 0.94, 1.19) and slightly lower for ear infections (RR = 1.17; 95% CI: 0.93, 1.46). Associations were slightly stronger among infants whose mothers spent more time at home during pregnancy. Air pollution exposure during the first year was highly correlated with prenatal exposure, so we were unable to discern the relative importance of each exposure period.

Conclusions: Our findings support the hypothesis that early-life exposure to ambient air pollution may increase the risk of upper and lower respiratory tract infections in infants.

Asthma, allergies, and respiratory infections are major health issues in childhood, and there is growing evidence that traffic-related air pollution is a risk factor for the development of asthmatic/allergic symptoms and respiratory infections (Braback and Forsberg 2009; Hoek et al. 2012). Ear infections are one of the leading causes of physician visits in young children as a major complication of a preceding upper respiratory infection, and they have been moderately associated with ambient air pollution (Brauer et al. 2006; Macintyre et al. 2011; Zemek et al. 2010). The first years of life are thought to be the most important period for the development of asthma and allergies (Landau 2006). Because both the respiratory and immune systems are still under development, exposure to air pollution during this period may be critical for lasting adverse effects on respiratory health (Bateson and Schwartz 2008).

Most studies investigating the effects of outdoor air pollution on respiratory health, allergic symptoms, and ear infections during early childhood have focused on postnatal exposure, and only some of them have applied air pollution modeling techniques to obtain individual estimates of exposure (Brauer et al. 2002; Gehring et al. 2002; Karr et al. 2009; Macintyre et al. 2011; Morgenstern et al. 2007). There is increasing evidence from experimental and epidemiologic studies that the prenatal period is a critical window for harmful effects of toxic chemicals on respiratory health early in life and beyond (Pinkerton and Joad 2006). However, the effect of air pollution exposure during pregnancy on respiratory health and allergic responses early in life has been examined in only a small number of studies; these differed considerably with regard to exposure assessment and outcome definitions, which included physician records of asthma diagnosis (Clark et al. 2010), lung function measurements (Latzin et al. 2009), parental reports of respiratory infections and wheezing (Jedrychowski et al. 2005, 2011), and symptoms such as cough, sore throat, runny nose, or difficult breathing (Jedrychowski et al. 2005, 2011; Miller et al. 2004). Only two studies have assessed eczema (Jedrychowski et al. 2011; Miyake et al. 2010), and only one examined associations according to the time period of exposure during pregnancy (Esplugues et al. 2011).

Our aim was to investigate, in a Spanish population-based birth cohort, associations of exposure to outdoor air pollution during pregnancy and during the first year of life with respiratory illness, eczema, and ear infections during the first 12–18 months of life.

## Methods

*Study population.* A population-based birth cohort was established between 2003 and 2008 in four Spanish regions (Asturias, Gipuzkoa, Sabadell, and Valencia) as part of the multicenter INMA-INfancia y Medio Ambiente [Environment and Childhood] project. Women were enrolled during the first routine prenatal care visit at the primary health care center or hospital, provided that they fulfilled the inclusion criteria (age ≥ 16 years, wish to deliver in the reference hospital, no communication problems, singleton pregnancy, and no assisted conception) (Guxens et al. 2011). Follow-up interviews were performed during the third trimester of pregnancy, and for infants at 6 and 18 months in Asturias, at 14 months in Gipuzkoa, at 6 and 14 months in Sabadell, and at 12 months in Valencia. The hospital ethics committees in the participating regions approved the study, and all women provided written informed consent before participation.

*Air pollution exposure assessment.* We developed area-specific land use regression (LUR) models of nitrogen dioxide (NO_2_) and benzene to estimate residential-based exposures during pregnancy and the first year of life. Ambient levels of NO_2_ and benzene were measured with Radiello passive samplers during several periods of one week each (Fundazione Salvatore Maugeri, Padua, Italy). Measurements taken in the different sampling campaigns were averaged to represent annual mean levels in each study area. Potential predictor variables such as traffic indicators, surrounding land uses, topography, and population density were derived in the geographic information system ArcGIS version 9.1 (ESRI, Redlands, CA, USA). Multiple linear regression models were built using a supervised forward stepwise procedure. Traffic-related variables, altitude, and land uses (urban, industrial, or agricultural) were the main predictor variables in the final LUR models. Models explained 52–75% of the variability in measured NO_2_ levels, and 44–73% of the variability in measured benzene levels, depending on the study area.

Residential addresses were geocoded using either commercial geocoding applications [in Asturias (BatchGeo; www.batchgeo.com) and Valencia (ViaMichelin; http://www.viamichelin.es)] or mapping applications from the regional governments [in Gipuzkoa (Government of Gipuzkoa; http://www.gipuzkoaaldundia.net) and Sabadell (Cartographic Institute of Catalonia; http://www.icc.cat)]. LUR models were applied to predict outdoor levels of both pollutants at the residential addresses. For women and infants who changed their residential address during the study period, we calculated an average exposure estimate weighted by the time spent at each residence. We derived individual exposures to NO_2_ and benzene during pregnancy by multiplying the LUR estimate by the ratio between the average concentrations measured at the fixed stations over the woman’s pregnancy period and over the whole air pollution sampling period. We applied the same procedure to estimate exposures during each trimester of pregnancy and the first year of life. A description of the sampling campaigns, LUR models, and monitoring stations used for temporal adjustment in each of the cohorts is given in Supplemental Material, Table S1 (http://dx.doi.org/10.1289/ehp.1205281).

*Definition of the outcomes.* Information on doctor-diagnosed lower respiratory tract infection (LRTI) and symptomatic wheezing, eczema, and ear infections were obtained through interviewer-led questionnaires at each follow-up. Occurrence of LRTI was defined as an affirmative answer to the question “Has a doctor told you that your son/daughter has had a chest infection?” An additional question referred to a specific doctor diagnosis of bronchitis, bronchiolitis, pneumonia, or pneumonitis. Parents were also asked whether the children had eczema, wheezing episodes, and ear infections from birth until follow-up. For these outcomes, parents were not specifically asked to report doctor-diagnosed conditions only. Due to funding constraints, the follow-up originally planned at 18 months in the Asturias cohort was actually performed at older ages (mean ± SD = 29.1 ± 5.7 months); but to keep the adherence to the common study protocol, mothers were specifically asked to refer to the period 6–18 months of age when answering questions.

*Covariates.* Potential confounding and effect modification variables (Table 1) were obtained through questionnaires administered during the first and third trimesters of pregnancy, at birth, and during infants’ follow-up. Cotinine levels were measured in a 24-hour sample of urine collected in the third trimester of pregnancy using the Cotinine 175 Micro-Plate EIA Kit (Ora Sure Technologies, Inc., Bethlehem, PA, USA).

**Table 1:** Child and parental characteristics of the study population by study area.

Characteristic	Asturias (n = 434)	Gipuzkoa (n = 540)	Sabadell (n = 521)	Valencia (n = 704)	Overall (n = 2,199)
Male sex	51.8	49.9	52.0	52.8	51.7
Birth season					
Winter	21.9	29.4	26.1	25.7	25.9
Spring	19.4	28.4	30.5	18.9	24.1
Summer	30.4	24.3	23.2	21.3	24.3
Fall	28.3	17.8	20.2	34.1	25.7
Low birth weight	5.4	3.9	4.6	5.4	4.9
Preterm birth	5.1	3.4	3.1	5.3	4.3
Siblings at birth	38.9	42.1	41.5	44.6	42.2
Duration of breastfeeding (weeks)					
None	27.5	11.0	6.7	15.8	14.7
1–15	29.2	19.2	25.8	26.0	24.9
16–24	16.6	16.5	16.3	14.6	15.9
> 24	26.7	53.3	51.2	43.6	44.6
Day care attendance	50.4	47.4	29.7	20.9	35.0
Non-Spanish mother	3.2	3.3	10.6	12.1	7.8
Prepregnancy body mass index ≥ 25 kg/m2	30.6	20.0	27.4	27.8	26.4
Maternal education					
Primary or less	17.7	13.5	27.4	31.5	23.4
Secondary	45.2	36.5	41.9	43.5	41.7
University	37.1	49.9	30.7	25.0	34.9
Maternal asthma	7.6	5.9	8.5	7.4	7.3
Paternal asthma	8.1	7.2	6.7	5.1	6.6
Maternal allergy	19.4	23.3	29.6	23.5	24.1
Paternal allergy	15.2	18.1	21.9	17.9	18.4
Maternal smoking during pregnancy	18.1	13.2	14.1	23.9	17.8
Secondhand smoke during pregnancya					
No	50.7	42.1	36.9	26.8	37.5
1 source	35.6	43.2	42.7	39.0	40.3
≥ 2 sources	13.7	14.7	20.4	34.2	22.2
Maternal postnatal smoking	26.1	19.4	25.4	31.3	26.0
Paternal postnatal smoking	33.2	31.5	41.5	45.8	38.9
Exposure to gas cooking during pregnancy	21.2	15.4	61.7	64.6	43.6
Mothers who spent ≥ 15 hr/day at home during pregnancy	91.7	42.5	52.5	62.8	60.9
Maternal fruits and vegetables intake during pregnancyb					
≤ 517.26 g/day	47.7	48.3	47.2	56.0	50.4
> 517.26 g/day	52.3	51.7	52.8	44.0	49.6
Maternal circulating vitamin D during pregnancyc					
≤ 20.89 ng/mL	39.1	36.6	38.2	23.0	33.2
20.90–30.20 ng/mL	36.1	35.8	28.9	34.2	33.7
> 30.20 ng/mL	24.8	27.7	32.9	42.7	33.1
Child’s age at latest follow-up (months)	29.2 ± 5.7	14.3 ± 1.2	14.5 ± 0.8	12.4 ± 1.1	16.59 ± 6.7
Maternal age at delivery (years)	33.0 ± 4.1	32.6 ± 3.6	31.6 ± 4.2	31.3 ± 4.4	32.01 ± 4.2
Cotinine levels in urine at 32 weeks of gestation (ng/mL), log scale	2.2 ± 3.1	2.0 ± 2.5	2.2 ± 2.8	3.0 ± 3.0	2.41 ± 2.9
Values are percent for categorical variables and mean ± SD for continuous variables. aSources of exposure to secondhand smoke reported during pregnancy: home, work, and other places. bMedian cut-off value. cTertile cut-off values.					

*Statistical analysis.* We first performed area-specific analyses, using log-binomial regression models to estimate relative risks of outcomes during the first 12–18 months of life associated with an increase of 10 µg/m^3^ and 1 µg/m^3^ in NO_2_ and benzene exposures, respectively, for the entire pregnancy, each trimester, and the first year of life in each of the four study areas. We tested a subset of *a priori* selected covariates (Table 1) for inclusion in the models using backward selection, and we retained only those covariates associated with *p* < 0.1 or that led to a change in the relative risk estimate of at least 10% when removed from the model. In a second step, we performed a meta-analysis using random-effects models combining the relative risk estimates from each area, and estimated *p*-values for the chi-square test for heterogeneity. As a comparative analysis, we performed additional models using a biological approach: We included all the *a priori* selected covariates regardless of their *p*-value or effect on the exposure–outcome association. We also estimated relative risks associated to an interquartile range (IQR) increase in NO_2_ and benzene exposure to be able to compare the effect of the two pollutants on health outcomes.

Because LUR estimates of prenatal exposure were based on women’s residential addresses, we performed a sensitivity analysis restricted to women who spent more time at home during pregnancy (≥ 15 hr/day), assuming that this subset of participants is less affected by exposure misclassification (Nethery et al. 2009). Similarly, for postnatal exposures we estimated associations in the subset of infants who were not attending child care at the time of follow-up, under the assumption that they spent more time at or around their residence. Additional sensitivity analyses were conducted excluding infants from the Asturias cohort (given its particularities regarding infant age at follow-up and the relevant contribution of industrial sources on air pollution levels), infants born preterm (< 37 weeks gestation), infants with low birth weight (< 2,500 g), and infants whose mothers smoked during pregnancy or after birth. Finally, we stratified by duration of breastfeeding (none, ≤ 6 months and > 6 months), maternal intake of fruits and vegetables during pregnancy (categorized using the median value), and maternal circulating vitamin D (categorized by tertiles) to examine potential effect modification by factors that may increase antioxidant and detoxification capacity, as suggested in a recent analysis of air pollution and neurodevelopment in the same cohort (Guxens et al. 2012).

Statistical analyses were performed using Stata version 10.1 (StataCorp, College Station, TX, USA).

## Results

From the 2,644 women enrolled in the study at the beginning of pregnancy, we obtained data on both air pollution exposure and health outcomes at 12–18 months for 83% (*n* = 2,199) of their infants [see Supplemental Material, Figure S1 (http://dx.doi.org/10.1289/ehp.1205281)].

Characteristics of the study population are presented in Table 1. Most women were nulliparous (58%), nonsmokers during the entire pregnancy (82%) but exposed to passive smoking (62.5%), and had at least secondary education (77%). The percentage of maternal smoking increased in the postnatal follow-up (26%) but remained lower than paternal smoking (39%). The percentage of missing data among the covariates ranged from 0 to 5.4% (data not shown). Compared with the 2,199 mother–child pairs included in the study, the 445 nonparticipating women had lower educational level, higher prevalence of smoking during pregnancy, less prevalence of allergy history, and were less likely to be Spanish (*p* < 0.10) (data not shown).

**Figure 1:**
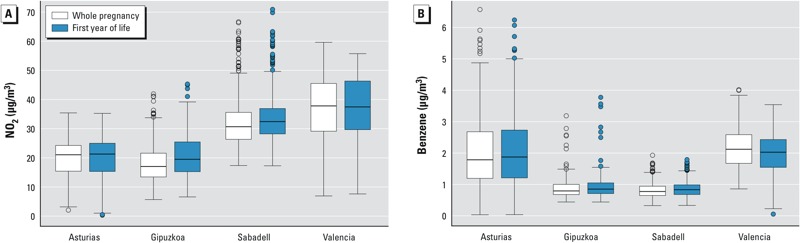
Distribution of exposures to outdoor NO_2_ (*A*) and benzene (*B*) during pregnancy and first year of life by study area. Boxes extend from the 25th to the 75th percentile, horizontal bars represent the median, whiskers extend 1.5 times the length of the IQR above and below the 75th and 25th percentiles, respectively, and outliers are represented as points.

About 37% of the infants had a diagnosis of LRTI during the first 12–18 months of life (Table 2), with almost all (98%) classified as bronchitis or bronchiolitis. Thirty-eight percent of mothers reported that their child had one or more symptomatic episodes of wheezing, 33% reported one or more ear infections, and 22% reported at least one episode of eczema. All health outcomes were more likely to have been reported for children in the Asturias cohort, which also had the oldest infants’ mean age at latest follow-up (mean ± SD = 29.2 ± 5.7 months compared with 13.6 ± 1.4 months for the three other cohorts combined). NO_2_ exposure estimates were higher in the predominantly urban Valencia and Sabadell cohorts, whereas benzene levels were highest in Asturias, probably due to the additional contribution of industrial emissions (Figure 1). The correlation between NO_2_ and benzene was considerably lower in Asturias (Spearman rho = 0.29) than in the other three areas (Spearman rho = 0.70 for all 3 combined). Levels of each pollutant were moderately to highly correlated between trimesters of pregnancy (Spearman rho = 0.62–0.79) and highly correlated between the entire prenatal period and the first year of life (Spearman rho = 0.94 for NO_2_ and 0.89 for benzene).

**Table 2:** Children reported to have had at least one episode of each health outcome during the first 12–18 months of age [*n* (%)].

Health outcome	Asturias (n = 434)	Gipuzkoa (n = 540)	Sabadell (n = 521)	Valencia (n = 704)	Overall (n = 2,199)
Doctor-diagnosed LRTI	180 (44.7)	211 (39.1)	206 (40.9)	204 (29.0)	801 (37.2)
Wheezing	242 (59.3)	195 (36.1)	186 (37.7)	181 (25.7)	804 (37.5)
Eczema	148 (38.6)	88 (16.4)	107 (22.1)	117 (16.7)	460 (21.9)
Ear infections	173 (43.1)	194 (36.1)	174 (35.0)	172 (24.4)	713 (33.3)

Unadjusted and adjusted associations between exposure to air pollutants and development of health outcomes based on random-effects meta-analyses are shown in Table 3. Relative risks (RRs) for a 10-µg/m^3^ increase in NO_2_ exposure and 1-µg/m^3^ increase in benzene exposure during the entire prenatal period suggest increased risk for LRTI and ear infections. The latter outcome is also positively associated with exposure to both pollutants during the first year of life. Relative risk estimates suggest a positive association between postnatal exposure to benzene and eczema, but the rest of RRs for this outcome are close to unity. There are no substantial differences between crude and adjusted effect estimates for any of the two pollutants. Models adjusted for all of the potential covariates identified *a priori* produced slightly higher RRs with wider 95% CIs (results not shown). Results for an IQR increase in NO_2_ and benzene exposure during the entire pregnancy showed similar associations for both pollutants with LRTI (RR = 1.08; 95% CI: 0.97, 1.21 for NO_2_, and RR = 1.06; 95% CI: 0.94, 1.19 for benzene), whereas the association with ear infections was slightly higher for NO_2_ (RR = 1.31; 95% CI: 0.97, 1.76) than for benzene (RR = 1.17; 95% CI: 0.93, 1.46) [see Supplemental Material, Table S2 (http://dx.doi.org/10.1289/ehp.1205281)].

**Table 3:** Associationa between prenatal and postnatal exposure to outdoor NO2 or benzene and doctor-diagnosed LRTI, wheezing, eczema, and ear infections during the first 12–18 months of age: results from random-effects meta-analysis across all four study areas.

Health outcome	Exposure period	NO2 (per 10-µg/m3 increase)					Benzene (per 1-µg/m3 increase)				
		Crude		Adjusted			Crude		Adjusted		
		RR	(95% CI)	RR	(95% CI)	p-Valueb	RR	(95% CI)	RR	(95% CI)	p-Valueb
Doctor-diagnosed LRTIc	Entire prenatal	1.04	(0.97, 1.11)	1.05	(0.98, 1.12)	0.685	1.06	(0.97, 1.15)	1.05	(0.96, 1.14)	0.814
	First trimester	1.05	(0.99, 1.11)	1.06	(1.00, 1.12)	0.570	1.06	(1.00, 1.13)	1.06	(0.99, 1.13)	0.527
	Second trimester	1.07	(1.01, 1.13)	1.08	(1.02, 1.15)	0.336	1.09	(1.00, 1.19)	1.10	(1.01, 1.20)	0.279
	Third trimester	1.00	(0.94, 1.05)	1.00	(0.93, 1.07)	0.233	1.01	(0.89, 1.15)	0.99	(0.87, 1.12)	0.100
	First year of life	1.01	(0.95, 1.08)	1.03	(0.95, 1.11)	0.259	1.04	(0.95, 1.13)	1.02	(0.93, 1.11)	0.608
Wheezingd	Entire prenatal	1.02	(0.94, 1.10)	1.03	(0.96, 1.10)	0.631	1.03	(0.90, 1.17)	1.01	(0.94, 1.09)	0.396
	First trimester	1.02	(0.96, 1.07)	1.02	(0.96, 1.09)	0.918	0.99	(0.93, 1.05)	1.00	(0.94, 1.06)	0.929
	Second trimester	1.04	(0.96, 1.13)	1.05	(0.99, 1.11)	0.568	1.05	(0.92, 1.20)	1.02	(0.96, 1.09)	0.404
	Third trimester	0.99	(0.92, 1.07)	1.01	(0.94, 1.08)	0.299	0.99	(0.93, 1.05)	1.00	(0.93, 1.07)	0.531
	First year of life	1.03	(0.95, 1.11)	1.04	(0.98, 1.12)	0.486	0.98	(0.90, 1.08)	0.97	(0.90, 1.05)	0.597
Eczemae	Entire prenatal	1.00	(0.90, 1.10)	1.00	(0.91, 1.10)	0.819	1.03	(0.93, 1.14)	1.02	(0.90, 1.16)	0.355
	First trimester	0.97	(0.89, 1.06)	0.97	(0.89, 1.05)	0.486	0.94	(0.82, 1.09)	0.93	(0.81, 1.08)	0.171
	Second trimester	1.00	(0.92, 1.09)	1.01	(0.92, 1.11)	0.332	1.02	(0.89, 1.17)	1.02	(0.86, 1.22)	0.109
	Third trimester	1.01	(0.92, 1.10)	1.01	(0.92, 1.10)	0.815	1.07	(0.97, 1.19)	1.07	(0.97, 1.18)	0.363
	First year of life	1.01	(0.92, 1.11)	1.02	(0.92, 1.12)	0.924	1.10	(1.00, 1.22)	1.09	(0.98, 1.21)	0.690
Ear infectionsf	Entire prenatal	1.14	(0.99, 1.33)	1.18	(0.98, 1.41)	0.002	1.13	(0.99, 1.28)	1.13	(0.95, 1.34)	0.121
	First trimester	1.07	(0.96, 1.20)	1.11	(0.99, 1.24)	0.048	1.06	(0.96, 1.17)	1.08	(1.02, 1.15)	0.726
	Second trimester	1.10	(0.95, 1.26)	1.16	(0.98, 1.37)	< 0.001	1.07	(0.91, 1.26)	1.13	(1.00, 1.27)	0.120
	Third trimester	1.09	(0.96, 1.24)	1.12	(0.98, 1.29)	0.008	0.99	(0.85, 1.15)	1.02	(0.92, 1.13)	0.228
	First year of life	1.14	(1.02, 1.28)	1.15	(1.01, 1.31)	0.036	1.10	(1.10, 1.20)	1.08	(0.99, 1.18)	0.984
aCrude associations are adjusted for child’s sex and age at follow-up. Adjusted associations also include the following covariates depending on the study area. bChi-square test for heterogeneity. cChild care attendance, siblings at birth, maternal asthma, parental allergy, maternal age at delivery, prepregnancy body mass index, and cotinine levels in urine at 32 weeks of gestation (log-transformed). dDuration of breastfeeding, child care attendance, siblings at birth, paternal allergy, parental asthma, maternal age at delivery, maternal education, prepregnancy body mass index, maternal smoking during pregnancy, paternal postnatal smoking, and cotinine at 32 weeks of gestation (log-transformed). eBirth season, child care attendance, paternal asthma, maternal allergy, maternal age at delivery, prepregnancy body mass index, maternal smoking during pregnancy, cotinine levels in urine at 32 weeks of gestation (log-transformed), and exposure to gas cooking during pregnancy. fBirth season, child care attendance, siblings at birth, parental asthma and allergy, prepregnancy body mass index, secondhand smoke exposure during pregnancy, and parental postnatal smoking.											

RRs for LRTI and ear infections in association with exposure to NO_2_ and benzene were slightly higher for exposure during the second trimester compared with other trimesters. Because most of the associations were adjusted by cotinine levels measured in urine at 32 weeks of pregnancy, which reflect very recent exposure to tobacco smoke, we ran additional models adjusted for questionnaire-based smoking variables instead, but associations remained essentially unchanged (data not shown).

Except for estimates for NO_2_ and ear infections, meta-analysis estimates did not show significant heterogeneity among the four study areas (*p* > 0.10; data not shown). Area-specific associations between NO_2_ and ear infections were positive for all time periods for all of the study areas except Sabadell [see Supplemental Material, Table S3 (http://dx.doi.org/10.1289/ehp.1205281)]. In general, associations between ear infections and benzene were stronger in the two areas characterized by higher exposure levels—Asturias and Valencia. Because swimming pool attendance was particularly high in the Sabadell cohort at 14 months of age (65% vs. 45% in the other cohorts), we stratified the analysis by this variable, but RRs for ear infections and NO_2_ were comparable in both groups (results not shown).

**Figure 2:**
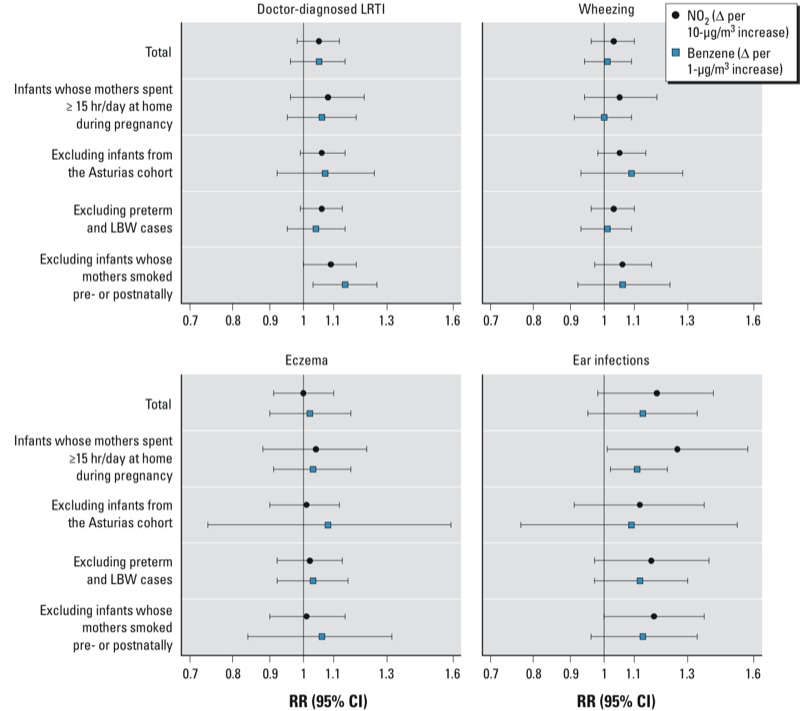
Adjusted associations from random-effects meta-analyses between exposure to outdoor NO_2_ or benzene during pregnancy and LRTI, wheezing, eczema, and ear infections at 12–18 months of age in several subsamples of the cohort. Models were adjusted for the covariates indicated in Table 3.

RRs for NO_2_ (but not benzene) were slightly higher for infants whose mothers spent ≥ 15 hr/day at home during pregnancy compared with the cohort as a whole (1.08 vs. 1.05 for LRTI, 1.05 vs. 1.03 for wheezing, 1.04 vs. 1.00 for eczema, and 1.26 vs. 1.18 for ear infections) (Figure 2). Excluding the Asturias cohort did not show meaningful differences for any of the outcomes and resulted in wider confidence intervals for all the RRs for benzene exposure. Associations were essentially unchanged when preterm births and low birth weight children were excluded, as well as after excluding infants whose mothers smoked during pregnancy or after birth. Restricting analyses to infants who were not attending child care at the time of follow-up did not produce any clear trend (RRs associated with postnatal exposure to NO_2_ decreased from 1.03 to 1.01 for LRTI, remained the same for wheezing, and increased from 1.02 to 1.04 for eczema and from 1.15 to 1.17 for ear infections; data not shown). Finally, we found no evidence of effect modification by maternal fruit and vegetable intakes, circulating vitamin D during pregnancy, or the duration of breastfeeding [see Supplemental Material, Table S4 (http://dx.doi.org/10.1289/ehp.1205281)].

## Discussion

In this prospective birth cohort study, we found associations with exposure to ambient air pollution during pregnancy and the first year of life with LRTI and ear infections during the first 12–18 months of life, although the high correlation between pre- and postnatal exposures did not allow us to identify whether either or both periods were driving the observed effects. We did not find clear or consistent evidence of associations between exposure to air pollution and eczema or wheezing symptoms. Associations were not substantially altered by adjusting for potential confounders. In general, associations were slightly stronger for air pollution exposure during the second trimester of pregnancy compared with other trimesters.

We found similar patterns in the associations for NO_2_ and benzene despite their moderate correlation. NO_2_ is a widely used marker of traffic-related air pollution, whereas benzene levels can reflect both traffic and industrial activities, depending on the characteristics of the study area. Rather than a potential causative agent by itself, benzene may be acting as a surrogate for a mixture of predominantly traffic-driven pollutants, similar to NO_2_.

Differences in the proportions of children reported to have one or more episodes of each outcome may reflect differences in the length of follow-up among the four areas, which ranged from 12 to 18 months. All outcomes were more common in Asturias than in the other study areas, possibly because mothers reported on illnesses through 18 months when their children were 29 months of age on average, which could have led to over-reporting of outcomes. Excluding infants from Asturias resulted in wider CIs, particularly for RRs associated with benzene exposure, but associations were not modified to any great extent.

A major strength of our study is the prospective design beginning in early pregnancy, which allowed us to collect individual data on health outcomes and potential confounders using similar protocols in the four study areas. An additional strength is the use of temporally adjusted LUR models to estimate individual exposures during specific time periods. However, despite their spatial accuracy, LUR estimates are still a proxy for personal exposure, which may be influenced by individual time–activity patterns (Nethery et al. 2009). Associations were slightly stronger when we limited analyses to infants whose mothers spent more time at home during pregnancy, both in the present study and in a previous analysis of birth outcomes (Estarlich et al. 2011), but we did not observe consistent changes in estimates after excluding infants attending child care at the time of follow-up.

The main limitation is that health data were obtained through questionnaires rather than clinical records. Recall bias and potential misclassification of the outcomes are possible, particularly in infants with less distinctive symptoms as compared with older children (Braback and Forsberg 2009).

Associations between NO_2_ and LRTI, and to a lesser extent wheezing, are consistent with positive associations previously reported for respiratory infections and wheezing among 2-year-old infants in other European areas with similar NO_2_ exposure levels (Brauer et al. 2002; Morgenstern et al. 2007). Wheezing at very young ages is highly associated with viral infections (Martinez et al. 1995), and many children who wheeze early in life have only a few episodes and do not wheeze at later ages. Nevertheless, even transient early wheeze is associated with asthma diagnosis later in childhood (Savenije et al. 2011).

Pre- and postnatal exposure to tobacco smoke is a risk factor for ear infections in early childhood, but evidence of an effect of ambient air pollution is inconclusive (Heinrich and Raghuyamshi 2004). Two recent studies reported modest but significant positive associations between short-term air pollution exposure and emergency department visits for ear infections (Zemek et al. 2010) and long-term exposure and physician diagnosis of otitis media (Macintyre et al. 2011). Similar to our study, a 10-µg/m^3^ increase in postnatal exposure to NO_2_ was associated with ear infections at 2 years of age in children from the Netherlands and Germany (Brauer et al. 2006).

Eczema is often the first manifestation of atopic disease during early childhood. Some of the factors that increase the risk of wheezing and respiratory infections in infants have less or no association with atopic eczema, which suggests differences in etiology between eczema and wheezing in infancy (Linneberg et al. 2006). Results from the few existing cohort studies on air pollution and eczema or allergic sensitization in young children are inconsistent, and comparisons are limited by differences in exposure assessment among studies (Brauer et al. 2002; Jedrychowski et al. 2011; Miyake et al. 2010; Morgenstern et al. 2008; Nordling et al. 2008). A Dutch cohort that also used LUR models to estimate individual NO_2_ exposures did not observe associations between doctor-diagnosed eczema at 2 years of age and NO_2_ (odds ratio = 0.96; 95% CI: 0.85, 1.08 for a 10-µg/m^3^ increase in NO_2_) (Brauer et al. 2002). The RRs close to unity for eczema in association with NO_2_ exposure in our study are consistent with their findings.

Several studies have also estimated associations between prenatal exposure to air pollution and respiratory health outcomes early in life. Infants from a Polish cohort whose mothers were exposed to higher levels of polycyclic aromatic hydrocarbons during pregnancy (measured through 48-hr personal monitoring) had more frequent and longer episodes of several respiratory symptoms during the first year of life, including ear infections and wheezing (Jedrychowski et al. 2005). Pre- and postnatal exposures to several air pollutants, including NO_2_, have been positively associated with asthma in preschool-age children (Clark et al. 2010). In a subset of 352 children from the present study, LUR estimates of NO_2_ exposure levels during pregnancy and home outdoor measurements of NO_2_ in the first year of life were not significantly associated with LRTI or with wheezing during the first year of life (Esplugues et al. 2011).

Air pollution exposures during pregnancy and first year of life tend to be highly correlated, which limits the interpretation of estimates from mutually adjusted models (Clark et al. 2010). In contrast with studies that characterize exposures based on measurements from nearest fixed monitoring stations (Mortimer et al. 2008), our exposure assessment approach emphasized spatial over temporal variation, which may have contributed to the very high correlations between pre- and postnatal exposures in our study. Results from the sensitivity analyses did not clarify the relative importance of each exposure period, because infants who did not attend child care were also more prone to be born to mothers who had spent more time at home during pregnancy. Therefore, it is not clear from our data which period (if any) was more relevant to health effects.

A pooled analysis of 12 studies on parental smoking and children’s respiratory health suggested independent effects of pre- and postnatal tobacco smoke exposures (Pattenden et al. 2006). However, associations between *in utero* exposure and respiratory outcomes in children appear to decline with age (Zlotkowska and Zejda 2005). From a biological perspective, both periods are likely to influence respiratory health given that lung development is a continuum of different phases that start in the embryogenesis and finish in early adolescence (Pinkerton and Joad 2006).

The exact mechanism whereby *in utero* exposure to ambient air pollution might increase the risk of postnatal respiratory infections is not known, but it has been hypothesized that air pollutants may affect the developing immune system which could increase susceptibility to virally induced infections after birth (Jedrychowski et al. 2005). We found slightly stronger associations with exposures during the second trimester of pregnancy, although differences must be interpreted with caution given the moderate to high correlations among trimester-specific exposures and the small differences in effect estimates. To our knowledge, apart from the study in the Valencia subsample of the INMA cohort (Esplugues et al. 2011), only one other cohort study of 232 asthmatic children assessed trimester-specific exposures during pregnancy in relation to allergic sensitization at 6–11 years of age (Mortimer et al. 2008). The authors reported stronger associations for exposures during the first and second trimester of pregnancy, which suggests that alterations of developmental steps in this time period could lead to a shift of the T helper type 1 (Th1)/T helper type 2 (Th2) balanc in the immune system toward increased Th2 responses during later fetal development and after birth (Prescott et al. 2003). Further research on the effect of air pollution exposure during specific developmental periods *in utero* may contribute to identify the relevant mechanism(s) involved.

## Conclusion

This study supports the hypothesis that *in utero* or early exposure to ambient air pollution may affect the development of the respiratory system and increase the risk of upper and lower respiratory tract infections. The high correlation between exposures during pregnancy and first year of life did not allow us to disentangle associations with pre- versus postnatal exposures. Further study of this birth cohort is needed to determine whether exposure to air pollution during the first years of life is associated with the development of asthma at later ages.

## Supplemental Material

(49 KB) PDF
