# Supplementary material for: Early-Life Exposure to Outdoor Air Pollution and Respiratory Health, Ear Infections, and Eczema in Infants from the INMA Study
Source: Environ Health Perspect. 2012 Dec 5;121(3):387–92. doi: 10.1289/ehp.1205281 (PMC3621204; doi:10.1289/ehp.1205281)
Supplement: (49 KB) PDF [file ehp.1205281.s001.pdf]

SUPPLEMENTAL MATERIAL

Early Life Exposure to Outdoor Air Pollution and Respiratory Health, Ear Infections, and Eczema in Infants from the INMA Study

Inmaculada Aguilera, Marie Pedersen, Raquel Garcia-Esteban, Ferran Ballester, Mikel Basterrechea, Ana Esplugues, Ana Fernández-Somoano, Aitana Lertxundi, Adonina Tardón, and Jordi Sunyer

Figure S1. Flowchart illustrating the main phases in the study………………………. p.2

Table S1. Air pollution sampling campaigns, LUR models and fixed stations used for temporal adjustment in each study area...…………………………………………….…..p.3

Table S2. Adjusted associations between prenatal exposure to outdoor NO_2 or benzene (per IQR increase) and LRTI, wheezing, eczema, and ear infections during the first 12-18 months of age……………………………………………………………… …p.6

Table S3. Adjusted associations between prenatal and postnatal exposure to outdoor NO_2 or benzene and ear infections by study area………………………..………….p.7

Table S4. Adjusted associations between prenatal exposure to outdoor NO_2 or benzene and LRTI, wheezing, eczema, and ear infections during the first 12-18 months of age, stratified by potential modifying factors…………………………………..………..p.9

Figure S1. Flowchart illustrating the main phases in the study

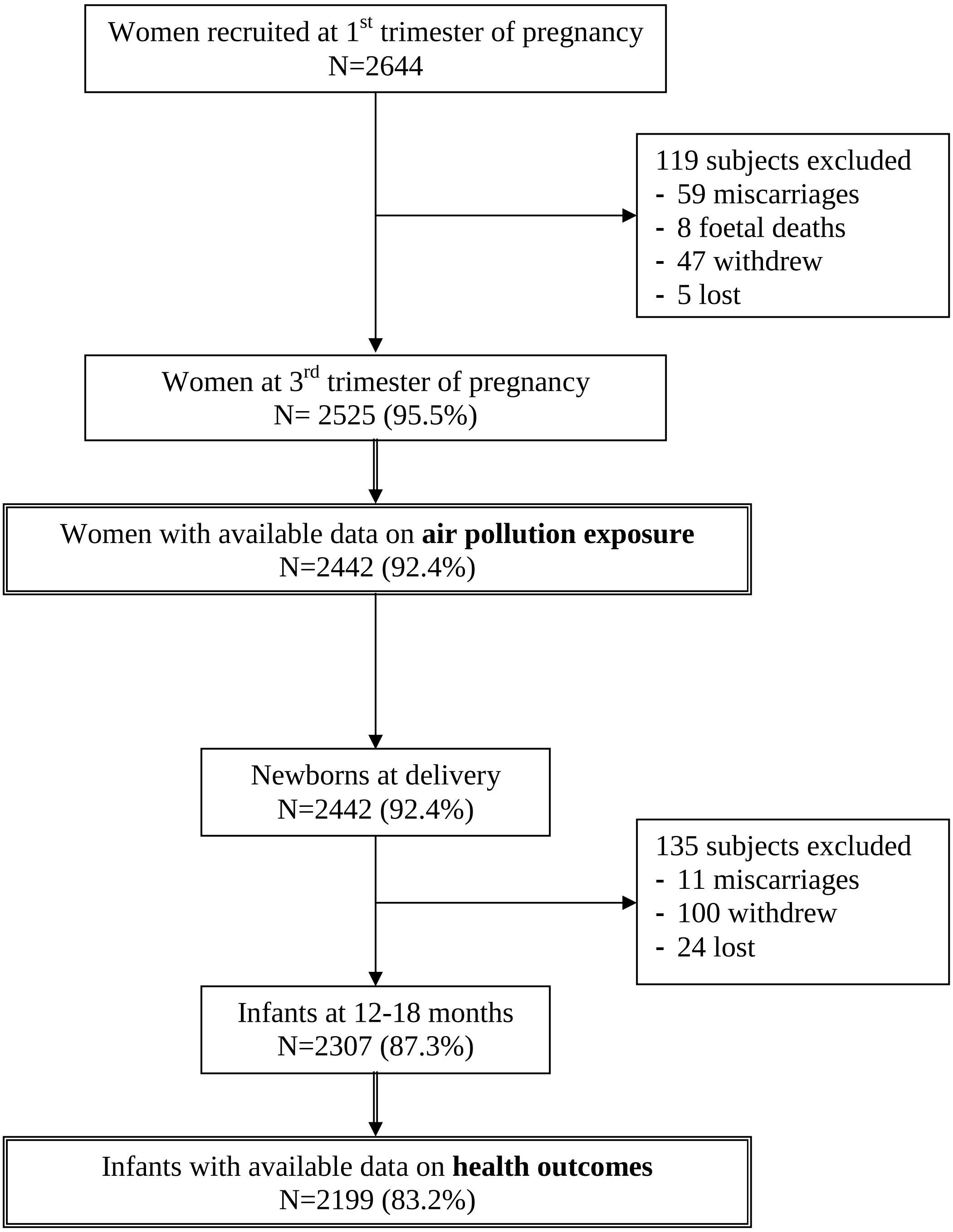

Table S1. Air pollution sampling campaigns, LUR models and fixed stations used for temporal adjustment in each study area

Study area	No. sampling sites	Sampling campaigns	Variables LUR model	R^2 LUR model	No. fixed stations (prenatal period)	No. fixed stations (postnatal period)	Pollutant used for temporal adjustment[a]
**NO_2**							
Asturias	67	June 05<br>November 05	Altitude<br>Log-transformed distance to nearest road<br>Agricultural or forest land cover within 300 m	0.52	4	4	NO_2
Gipuzkoa	85	February 07<br>June 07	Altitude (3 cat)<br>Valley factor<br>Distance to nearest major road (defined as ADT[b]>20,000)<br>Urban land cover within 100 m<br>Industrial land cover within 300 m	0.51	2	2	NO_2
Sabadell	57	April 05<br>June 05<br>October 05<br>March 06	Altitude<br>Urban and industrial land cover within 500 m<br>Road type (3 cat)	0.75	1	1	NO_2
Valencia	93	April 04<br>June 04<br>November 04<br>February 05	Log-transformed distance to nearest major road (defined as ADT[b]>10000)<br>Kriging estimate[c]<br>Industrial or urban land cover within 500 m	0.73	7	3	NO_2

Table S1 (continued)

**Study area**	**No. sampling sites**	**Sampling campaigns**	**Variables LUR model**	**R^2 LUR model**	**No. fixed stations (prenatal period)**	**No. fixed stations (postnatal period)**	**Pollutant used for temporal adjustment[a]**
**BENZENE**							
Asturias	67	June 05<br>November 05	Altitude<br>Distance to nearest road with ADT[b]=1,001-5,000<br>Continuous urban land cover within 300 m<br>Discontinuous urban land cover within 1,000 m<br>Agricultural or forest land cover within 1,000 m	0.73	4	4	SO_2
Gipuzkoa	85	February 07<br>June 07	Valley factor<br>Log-transformed distance to nearest major road (defined as ADT[b] >20,000)<br>Urban land cover within 100 m<br>Distance to industry	0.44	2	2	NO_2
Sabadell	57	April 05<br>June 05<br>October 05<br>March 06	Road type (3 cat)<br>Population density within 50 m<br>Urban land cover within 300 m<br>Building density within 500 m	0.73	1	1	NO_2
Valencia	93	April 04<br>June 04<br>November 04<br>February 05	Urban land cover within 500 m<br>Log-transformed distance to nearest major road (defined as ADT[b] >50,000)<br>Longitude	0.44	7	3	NO

[a] Due to the lack of benzene measurements in many stations and high missing data in those stations measuring benzene, we used the pollutant that exhibited the highest correlation with benzene for temporal adjustment
[b] ADT: Average daily traffic (vehicles/day)
[c] Mean of estimated NO_2 from kriging among campaigns

References:

- Aguilera et al. Estimation of outdoor NO_x, NO_2, and BTEX exposure in a cohort of pregnant women using land use regression modeling. Environ Sci Technol 42:815-821.
- Fernández-Somoano et al. Outdoor NO_2 and benzene exposure in the INMA (Environment and Childhood) Asturias cohort (Spain). Atmos Env 2011; 45:5240-5246.
- Iñiguez et al. Estimation of personal NO_2 exposure in a cohort of pregnant women. Sci Total Environ 2009; 407:6093-6099.

Table S2. Adjusted associations[a] between prenatal exposure to outdoor NO_2 or benzene (per IQR increase) and LRTI, wheezing, eczema, and ear infections during the first 12-18 months of age

		NO_2 (per IQR increase)			Benzene (per IQR increase)		
**Health outcome**	**Exposure period**	**IQR (μg/m^3)**	**RR**	**(95% C.I.)**	**IQR (μg/m^3)**	**RR**	**(95% C.I.)**
**Doctor-diagnosed LRTI**	Entire prenatal	16.5	1.08	(0.97, 1.21)	1.3	1.06	(0.94, 1.19)
	First trimester	18.6	1.11	(1.00, 1.24)	1.3	1.08	(0.99, 1.17)
	Second trimester	17.0	1.14	(1.03, 1.26)	1.0	1.10	(1.01, 1.20)
	Third trimester	17.5	0.99	(0.87, 1.12)	1.1	0.99	(0.86, 1.13)
	First year of life	15.7	1.04	(0.92, 1.17)	1.2	1.02	(0.92, 1.13)
**Wheezing**	Entire prenatal	16.5	1.05	(0.94, 1.18)	1.3	1.01	(0.92, 1.11)
	First trimester	18.6	1.04	(0.93, 1.17)	1.3	1.00	(0.93, 1.07)
	Second trimester	17.0	1.09	(0.99, 1.20)	1.0	1.02	(0.96, 1.09)
	Third trimester	17.5	1.01	(0.90, 1.14)	1.1	1.00	(0.93, 1.07)
	First year of life	15.7	1.07	(0.96, 1.19)	1.2	0.97	(0.88, 1.07)
**Eczema**	Entire prenatal	16.5	1.00	(0.85, 1.18)	1.3	1.02	(0.87, 1.21)
	First trimester	18.6	0.94	(0.80, 1.10)	1.3	0.92	(0.76, 1.11)
	Second trimester	17.0	1.02	(0.87, 1.19)	1.0	1.02	(0.86, 1.22)
	Third trimester	17.5	1.02	(0.87, 1.19)	1.1	1.08	(0.97, 1.20)
	First year of life	15.7	1.03	(0.88, 1.20)	1.2	1.10	(0.97, 1.26)
**Ear infections**	Entire prenatal	16.5	1.31	(0.97, 1.76)	1.3	1.17	(0.93, 1.46)
	First trimester	18.6	1.22	(0.99, 1.50)	1.3	1.11	(1.03, 1.20)
	Second trimester	17.0	1.29	(0.97, 1.71)	1.0	1.13	(1.00, 1.27)
	Third trimester	17.5	1.23	(0.96, 1.57)	1.1	1.02	(0.91, 1.14)
	First year of life	15.7	1.24	(1.01, 1.52)	1.2	1.10	(0.99, 1.22)

[a] Associations are adjusted for the covariates indicated in Table 3 of the main manuscript

Table S3. Adjusted associations between prenatal and postnatal exposure to outdoor NO_2 or benzene and ear infections by study area

	NO_2 (per 10 µg/m^3 increase)		Benzene (per 1 µg/m^3 increase)	
**Study area**	**RR**	**(95% C.I.)**	**RR**	**(95% C.I.)**
	Exposure during the entire prenatal period			
Asturias[a]	1.40	(1.14, 1.71)	1.10	(1.01, 1.21)
Gipuzkoa[b]	1.22	(1.01, 1.48)	0.87	(0.54, 1.40)
Sabadell[c]	0.92	(0.80, 1.06)	0.90	(0.57, 1.43)
Valencia[d]	1.25	(1.10, 1.42)	1.38	(1.12, 1.71)
**Meta-analysis**	1.18	(0.98, 1.41)	1.13	(0.95, 1.34)
p-Value[e]	0.002		0.121	
	Exposure during 1st trimester of pregnancy			
Asturias[a]	1.27	(1.06, 1.51)	1.08	(1.00, 1.16)
Gipuzkoa[b]	1.16	(0.97, 1.39)	0.87	(0.55, 1.36)
Sabadell[c]	0.96	(0.86, 1.09)	1.00	(0.67, 1.50)
Valencia[d]	1.13	(1.03, 1.25)	1.11	(1.00, 1.23)
**Meta-analysis**	1.11	(0.99, 1.24)	1.08	(1.02, 1.15)
p-Value[e]	0.048		0.726	
	Exposure during 2nd trimester of pregnancy			
Asturias[a]	1.34	(1.12, 1.59)	1.11	(1.01, 1.21)
Gipuzkoa[b]	1.18	(0.98, 1.42)	0.85	(0.53, 1.36)
Sabadell[c]	0.94	(0.84, 1.05)	0.92	(0.62, 1.36)
Valencia[d]	1.25	(1.13, 1.39)	1.24	(1.12, 1.37)
**Meta-analysis**	1.16	(0.98, 1.37)	1.13	(1.00, 1.27)
p-Value[e]	<0.001		0.120	
	Exposure during 3rd trimester of pregnancy			
Asturias[a]	1.34	(1.12, 1.61)	1.10	(1.00, 1.21)
Gipuzkoa[b]	1.20	(0.99, 1.45)	0.85	(0.53, 1.38)
Sabadell[c]	0.94	(0.83, 1.06)	0.91	(0.60, 1.37)
Valencia[d]	1.11	(1.01, 1.22)	0.96	(0.85, 1.08)
**Meta-analysis**	1.12	(0.98, 1.29)	1.02	(0.92, 1.13)
p-Value[e]	0.008		0.228	
	Exposure during the first year of life			
Asturias[a]	1.34	(1.11, 1.62)	1.08	(0.98, 1.20)
Gipuzkoa[b]	1.23	(1.03, 1.46)	1.04	(0.74, 1.47)
Sabadell[c]	0.97	(0.84, 1.12)	1.01	(0.64, 1.59)
Valencia[d]	1.14	(1.01, 1.29)	1.10	(0.89, 1.37)
**Meta-analysis**	1.15	(1.01, 1.31)	1.08	(0.99, 1.18)
p-Value[e]	0.006		0.120	

Adjusted for:

[a] Child's sex, age at follow-up, day care attendance, siblings at birth, and maternal asthma.

[b] Child's sex, age at follow-up, day care attendance, parental allergy, exposure to secondhand smoke during pregnancy, and birth season.

[c] Child's sex, age at follow-up, day care attendance, siblings at birth, and maternal post-natal smoking.

[d] Child's sex, age at follow-up, day care attendance, parental asthma, maternal pre-pregnancy BMI, and paternal post-natal smoking.

[e] *p*-Value for the Chi-square test for heterogeneity

Table S4. Adjusted associations[a] between prenatal exposure to outdoor NO_2 or benzene and LRTI, wheezing, eczema, and ear infections during the first 12-18 months of age, stratified by potential modifying factors.

	NO_2 (per 10 μg/m^3 increase)			Benzene (per 1 μg/m^3 increase)		
**Health outcome**	**RR**	**(95% C.I.)**	***p*-Value[b]**	**RR**	**(95% C.I.)**	***p*-Value[b]**
**Doctor-diagnosed LRTI**						
Fruits and vegetables intake during pregnancy						
≤ 517.26 gr/day	0.94	(0.85, 1.04)		1.07	(0.94, 1.23)	
> 517.26 gr/day	1.13	(1.04, 1.23)	0.094	1.10	(0.93, 1.29)	0.498
Circulating vitamin D during pregnancy						
≤ 20.89 ng/mL	1.11	(0.99, 1.26)		1.11	(0.84, 1.47)	
20.90 - 30.20 ng/mL	1.10	(0.94, 1.28)		1.24	(1.10, 1.40)	
> 30.20 ng/mL	0.99	(0.88, 1.12)	0.492	1.00	(0.84, 1.21)	0.041
Duration of breastfeeding						
None	1.05	(0.88, 1.25)		1.08	(0.84, 1.39)	
≤ 6 months	1.12	(0.94, 1.33)		1.03	(0.84, 1.25)	
> 6 months	0.89	(0.74, 1.08)	0.600	0.86	(0.59, 1.25)	0.537
**Wheezing**						
Fruits and vegetables intake during pregnancy						
≤ 517.26 gr/day	0.99	(0.88, 1.11)		1.04	(0.91, 1.18)	
> 517.26 gr/day	1.04	(0.95, 1.14)	0.675	1.02	(0.88, 1.19)	0.925
Circulating vitamin D during pregnancy						
≤ 20.89 ng/mL	1.06	(0.94, 1.20)		1.09	(0.85, 1.39)	
20.90 - 30.20 ng/mL	1.03	(0.90, 1.18)		1.07	(0.96, 1.19)	
> 30.20 ng/mL	0.98	(0.84, 1.15)	0.922	0.96	(0.76, 1.20)	0.416
Duration of breastfeeding						
None	1.05	(0.91, 1.22)		1.01	(0.90, 1.13)	
≤ 6 months	1.00	(0.89, 1.12)		0.95	(0.77, 1.17)	
> 6 months	1.13	(0.95, 1.34)	0.531	1.27	(0.97, 1.67)	0.182

Table S4 (continued)

Health outcome	NO_2 (per 10 μg/m^3 increase)			Benzene (per 1 μg/m^3 increase)		
	**RR**	**(95% C.I.)**	***p*-Value[b]**	**RR**	**(95% C.I.)**	***p*-Value[b]**
**Eczema**						
Fruits and vegetables intake during pregnancy						
≤ 517.26 gr/day	1.00	(0.87, 1.16)		1.08	(0.91, 1.28)	
> 517.26 gr/day	1.02	(0.90, 1.16)	0.981	1.12	(0.95, 1.32)	0.588
Circulating vitamin D during pregnancy						
≤ 20.89 ng/mL	0.97	(0.82, 1.16)		1.06	(0.88, 1.29)	
20.90 - 30.20 ng/mL	0.98	(0.81, 1.17)		0.98	(0.83, 1.16)	
> 30.20 ng/mL	0.98	(0.81, 1.19)	0.354	1.05	(0.83, 1.34)	0.876
Duration of breastfeeding						
None	0.99	(0.71, 1.38)		0.95	(0.45, 2.04)	
≤ 6 months	1.25	(0.86, 1.81)		1.19	(0.98, 1.46)	
> 6 months	0.97	(0.64, 1.48)	0.077	1.16	(0.76, 1.78)	0.135
**Ear infections**						
Fruits and vegetables intake during pregnancy						
≤ 517.26 gr/day	1.22	(1.00, 1.48)		1.15	(0.94, 1.39)	
> 517.26 gr/day	1.13	(0.95, 1.34)	0.655	1.15	(1.04, 1.28)	0.954
Maternal circulating vitamin D during pregnancy						
≤ 20.89 ng/mL	1.12	(0.88, 1.42)		1.11	(0.95, 1.29)	
20.90 - 30.20 ng/mL	1.29	(1.03, 1.60)		1.12	(0.99, 1.26)	
> 30.20 ng/mL	1.13	(0.88, 1.46)	0.294	1.31	(1.01, 1.70)	0.534
Duration of breastfeeding						
None	1.20	(0.98, 1.47)		1.19	(0.90, 1.59)	
≤ 6 months	1.21	(0.95, 1.53)		1.15	(0.88, 1.51)	
> 6 months	1.30	(0.98, 1.72)	0.906	1.31	(1.10, 1.56)	0.185

[a] Associations are adjusted for the covariates indicated in Table 3 of the main manuscript
[b] Combined *p*-value of the area-specific interaction term *p*-values
